# PedXylo: an integrated platform for autosomal and sex-linked pedigree analysis

**DOI:** 10.1186/s12711-026-01101-5

**Published:** 2026-11-09

**Authors:** G. Kövér, I. Nagy, A. T. Nguyen, J. Farkas, J. Sölkner, L. Vostry, I. Curik, D. Hršak

**Affiliations:** 1 Hungarian University of Agriculture and Life Sciences - Institute of Animal Sciences (MATE), Kaposvár, Hungary; 2 BOKU University, Vienna, Austria; 3 Czech University of Life Science Prague, Prague, Czechia; 4 Faculty of Agriculture, University of Zagreb, Zagreb, Croatia; 5 Ruđer Bošković Institute, Zagreb, Croatia

## Abstract

**Background:**

Pedigree-based inference remains central to animal breeding and to conservation, evolutionary, and human-isolate research. However, existing pedigree software offers no integrated support for X-linked analysis, limited partial-decomposition functionality, no standardised pedigree-quality framework, and limited scalability to industry-sized pedigrees.

**Results:**

We developed PedXylo, which extends previously published gene-dropping software for ancestral and partial inbreeding analysis into a comprehensive pedigree-analysis platform with integrated pedigree management, quality assessment, and effective-population-size estimation. PedXylo computes classical (F), Kalinowski’s new (F_N_), and ancestral (F_aB_, F_aK_, AHC) inbreeding coefficients on both autosomes and the X chromosome and provides ancestor-resolved (partial) decomposition of all five coefficient families in both inheritance modes, a combination not currently available in any other tool. Pedigree quality is summarised through complete (CGE) and discrete (DGE) generation equivalents alongside two new metrics: the asymmetrical pedigree equivalents (cAPE and mAPE) and the pedigree tree imbalance (PTI). cAPE and mAPE were only weakly correlated with CGE in the European bison reference population, indicating that they capture information not represented by depth-based metrics alone. Per-individual rates of inbreeding (ΔF and ΔF_X_) and effective population sizes are estimated for both autosomes and the X chromosome. We illustrate these analyses using the Habsburg lineage and a long-term captive European bison pedigree and demonstrate scalability on a 23.2-million-individual Holstein pedigree analysed in approximately 18 h on a single 40-thread workstation.

**Conclusions:**

PedXylo provides a unified, scalable framework for pedigree-based inference suitable for routine application in animal breeding and applicable to wide use in conservation, evolutionary, and human-isolate research. By integrating sex-linked analysis, partial decomposition of ancestral coefficients, and new pedigree-quality metrics, it enables hypotheses that could not previously be tested with available pedigree software. The software is freely available with documentation and worked examples.

## Background

Pedigree records have long provided a fundamental framework for understanding how genetic variation is transmitted across generations. Originating in human genealogical traditions, including the extensively documented pedigrees of European royal dynasties (e.g. [1]), , and in the stud books and herd books established for livestock breeding [2, 3], pedigree systems became central to the emerging science of genetics [4] alongside the rediscovery of Mendelian inheritance and the development of Mendelian genetics. During the twentieth century, pedigree analysis expanded substantially as formal methods were developed for estimating relatedness, inbreeding, and effective population size (Ne), providing a quantitative framework for understanding genetic variation and demographic processes in populations. Pedigree-based approaches have since become indispensable in the conservation of endangered domesticated [5] and wild species [6], the management of captive zoo populations [7, 8], the study of evolutionary and ecological processes in natural populations [9], and the investigation of genetic structure in isolated human populations [10].

Despite the rapid expansion of high-throughput genomic technologies, pedigree information remains essential for several reasons. First, pedigrees define the genealogical expectations of identity-by-descent, founder contributions, and inbreeding accumulation over generations — expectations against which genomic estimates can be evaluated and interpreted [11–13]. Second, pedigree records are often available for far larger numbers of individuals and over longer time spans than molecular datasets, enabling the reconstruction of demographic and genetic processes at temporal scales that are difficult to achieve with genomic data alone [9]. This is particularly true in animal breeding, where deep recorded pedigrees of tens of millions of individuals are routinely available and represent an information resource at a scale that current genotyping cannot yet match. Third, anomalies or unexpected patterns revealed by molecular analyses frequently require genealogical context for their interpretation, which is provided by pedigree analysis [11, 12]. Pedigrees and genomic data are therefore complementary rather than interchangeable, and the continued development of pedigree-based analytical tools remains an important priority.

However, the increasing size and complexity of modern pedigree datasets present substantial analytical challenges that are not fully addressed by existing software. Several tools are available for pedigree analysis, but each cover only a subset of the required analytical tasks. ENDOG [14] provides a broad set of autosomal inbreeding and population-structure statistics, and its current version (v4.8) also computes partial inbreeding coefficients with respect to specific ancestors as well as inbreeding restricted to a user-defined number of recent generations; however, it does not implement ancestral inbreeding coefficients. RelaX2 [15] is a command-based program designed for efficient handling of very large pedigrees, supporting pedigree checking, pruning, and the calculation of inbreeding and relationship coefficients, but does not provide ancestral decompositions. The kinship2 R package [16] is widely used for pedigree visualization and kinship computation in human genetics but lacks gene-dropping functionality, and the nadiv R package [17] constructs additive, dominance, and epistatic relatedness matrices for animal-model analyses in ecology and evolutionary biology but does not address inbreeding decomposition or pedigree-quality assessment. PyPedal [18] and PMx [8] offer additional capabilities but have not been updated to meet current computational requirements or to accommodate the full range of inbreeding decompositions now available. Retriever and Pointer [19] combine pedigree-based monitoring with stochastic simulation of genetic management for small captive populations but are limited to autosomal inheritance and do not implement ancestral or partial inbreeding coefficients. Pedig [20] is a Fortran package suited to very large populations, providing routines for inbreeding, probabilities of gene origin, relationship coefficients, and characterisation of pedigree information; several of its core routines are shared with our previous software, GRain [21, 22], which introduced gene-dropping-based estimation of ancestral and partial inbreeding coefficients but was limited to autosomal inheritance and did not include integrated pedigree-quality assessment. Critically, analytical implementations for sex-linked inheritance remain absent from pedigree-analysis software used in animal breeding and conservation, with only the classical X-linked inbreeding coefficient available in specialised human-genetics tools (e.g., the ribd [23] R package), despite the biological relevance of X-linked autozygosity for understanding genetic load, sex-specific selection, and purging [24, 25]. Standardised metrics for assessing pedigree quality, including structural balance and asymmetry, are likewise not routinely available, even though the reliability of inbreeding estimates depends strongly on pedigree depth and completeness. In addition, the per-individual rate of inbreeding (ΔF), which corrects classical F for pedigree depth and has been shown to detect inbreeding depression with greater sensitivity than F itself [26, 27], is directly provided as output by ENDOG and RelaX2 for autosomal inheritance; however, no equivalent X-linked quantity (ΔF_X_) is available in any existing pedigree software.

To address these limitations, we developed PedXylo, an integrated platform for comprehensive pedigree analysis that combines pedigree data management and error detection, quantitative assessment of pedigree quality, and estimation of autosomal and sex-linked genetic parameters within a single analytical workflow. PedXylo introduces three principal innovations: (i) the extension of all classical, new, ancestral, and partial inbreeding coefficients to X-linked inheritance, enabling joint autosomal and sex-linked analysis for the first time in publicly available software, together with direct output of the per-individual rate of inbreeding for both autosomes (ΔF) and the X chromosome (ΔF_X_), following the depth-corrected formulation of Gutiérrez et al. [27]; (ii) new pedigree-quality metrics, including the asymmetrical pedigree equivalent (APE) and pedigree tree imbalance (PTI), which quantify the structural balance of maternal and paternal ancestral contributions, as well as topological balance; and (iii) direct estimation of X-linked effective population size (Ne_X_) from pedigree data. In addition, PedXylo integrates established autosomal parameters, including classical, ancestral, and partial inbreeding coefficients and realised effective population size, and provides summary statistics and graphical outputs suitable for scientific publications and analytical reports. The platform is implemented in Rust for high computational performance and is available as both a command-line tool for large-scale analyses and a graphical user interface for interactive use. PedXylo is designed for a broad spectrum of applications spanning animal breeding, conservation biology, quantitative genetics in wild and managed populations, and human genetics.

## PedXylo software platform

### Overview and analytical workflow

PedXylo is designed to accommodate users with varying levels of computational expertise, from experienced programmers to researchers with limited informatics background.

A typical PedXylo analysis proceeds through three stages. In the first stage, the software loads pedigree data, performs integrity checks – including detection of parental sex inconsistencies and ancestry loops – and, if requested, partitions the pedigree into disconnected components (referred to as clans) or restricts the analysis to a user-defined subset of individuals based on year of birth (YOB) or an externally supplied reference population list. In the second stage, PedXylo performs gene-dropping simulations to propagate hypothetical alleles through the pedigree under either autosomal or X-chromosomal inheritance rules. In the third stage, the software computes all individual-level inbreeding coefficients, pedigree quality metrics, and effective population size estimates, and exports both tabular and graphical outputs. If structural errors are detected during loading, execution is halted and the user is informed accordingly.

### Gene-dropping framework

PedXylo builds on the gene-dropping framework of PEDIG [20], an early version of which is implemented in previous versions of GRain [21, 22]. Gene dropping is a Monte Carlo approach for pedigree analysis in which hypothetical alleles are assigned to pedigree founders and then transmitted through the pedigree according to Mendelian inheritance. The gene-dropping method was formalised for pedigree analysis by MacCluer et al. [28], building on earlier computer simulations of Mendelian inheritance [29]. In PedXylo, each simulation replicate begins by assigning a unique pair of alleles to every founder. These alleles are then propagated stochastically through subsequent generations, and self-encounters – instances in which an individual inherits two copies of the same founder allele – are recorded during the simulation. Under autosomal inheritance, each parent transmits one of its two alleles to an offspring with equal probability. Under X-chromosomal inheritance, however, males always transmit their single X-linked allele to daughters and never to sons, reflecting the hemizygous inheritance pattern of the X chromosome.

In summary, all identity-by-descent-based parameters; the classical, new, ancestral, and partial inbreeding coefficients and their X-linked counterparts are estimated exclusively through gene dropping, following GRain [21], in which gene dropping was adopted because it enables unbiased estimation of the ancestral inbreeding coefficients [30]. The derived quantities (ΔF, ΔF_X_, Ne, Ne_X_) combine these estimates with the complete generation equivalent (CGE), whereas all pedigree-quality metrics (CGE, MaxG, NKA, GPC, APE, PTI) and the generation interval [31] are computed deterministically from the pedigree structure, without simulation.

### Implementation and user interface

PedXylo was implemented in Rust because of its high performance, memory safety, and efficient handling of computational resources. Users may either compile the software locally using the Rust toolchain (rustup and cargo) or run operating system–specific precompiled binaries distributed with the software. Two versions are provided: a command-line (CL) version and a graphical user interface (GUI) version. The CL version is designed for large pedigrees, automated workflows, and high-performance computing environments, whereas the GUI version is intended for use on personal computers and is particularly suitable for small to moderate pedigrees. In the CL version, the user specifies all required commands at runtime and the workflow is executed non-interactively. In contrast, the GUI version enables interactive control of the workflow and outputs through buttons and input fields, both before and after the gene dropping simulation.

In the GUI version, for example, users can inspect and verify the pedigree data loaded into memory by selecting the Display pedigree option, which opens a dedicated window showing the pedigree records together with their corresponding column names. PedXylo is free and open-source software, released under the GNU General Public License v3.0; the source code, precompiled binaries, documentation, and worked examples are freely available from the GitLab repository at https://www.gitlab.com/dhrsak/pedxylo.git.

### Input data

The software accepts pedigree files in comma-separated value (CSV), space-delimited text (TXT), and tab-separated value (TSV) formats, with or without a header row. The required columns are individual ID, father ID, and mother ID; year of birth, date of birth and sex are optional. Year of birth is accepted as an integer cohort marker; while date of birth is accepted strictly in format YYYY-MM-DD, the latter being particularly useful for species with generation intervals shorter than one year (e.g. mice).

**Fig. 1:**
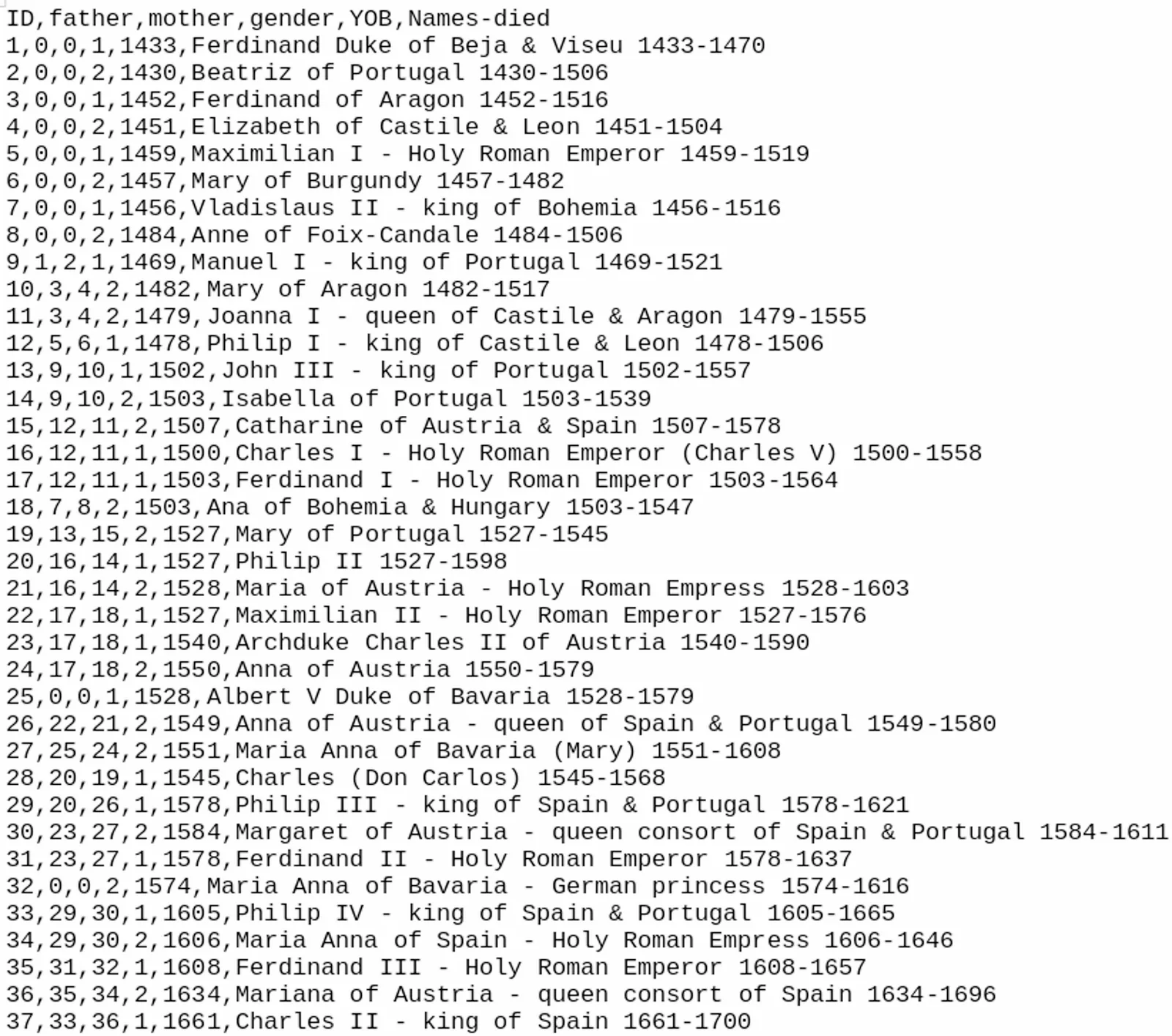
Habsburg pedigree input example. Example pedigree input file used for the Habsburg pedigree analysis (37 members). The flat-file input contains columns specifying individual ID, father ID, mother ID, gender (1 = male, 2 = female), year of birth (YOB), and individual name with lifespan. Individuals with both parents coded as 0 are founders and define the founder reference population (FRP)

**Fig. 2:**
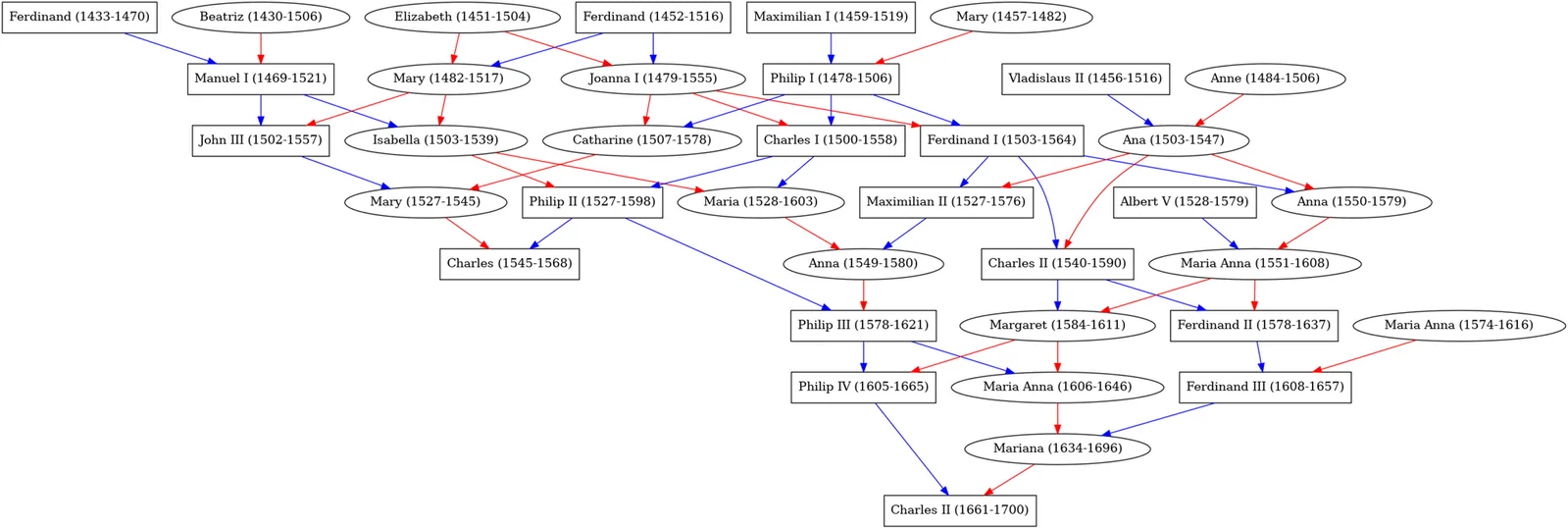
Habsburg family pedigree. Graphical representation of the Habsburg family pedigree. Blue arrows specify the transmission of male gametes, while red arrows specify the transmission of female gametes

Full details on input specifications, column naming conventions, and data coding are provided in the user manual and the README file. Figure 1 illustrates the input format using the Habsburg family pedigree (37 individuals born between 1430 and 1661). This pedigree is also presented graphically in Fig. 2 and serves as the basis for the application example in Sect.  Habsburg family pedigree; the reader may therefore use Fig. 1 to identify founders (individuals with unknown parents, coded as 0), to trace the genealogical relationships and transmission paths depicted in Fig. 2, and to understand how the concepts of founder reference population (FRP) and target reference population (TRP) defined in Sect. User-defined founder reference population apply to a real dataset.

### Simulation precision

The precision of gene-dropping-based estimates depends on the number of simulation replicates. Following previous versions of GRain, we recommend a minimum of one million (10^6^) gene drops as a practical compromise between computational demand and precision. Owing to the computational efficiency of PedXylo, however, up to one billion (10^9^) gene drops can be performed, yielding near-stable estimates with a precision up to five decimal places, except in extremely large pedigrees (see also Sect.  Partial inbreeding coefficients). PedXylo assesses this precision automatically: for every group of two or more full siblings among the analysed individuals, whose expected pedigree-based coefficients are identical by construction, the program computes the within-group standard deviation of each estimated autosomal coefficient and reports the results in a dedicated sibling quality-control output file (see Sect.  Autosomal and sex-linked inbreeding coefficients for an example). Any dispersion within a sibling group reflects only Monte Carlo sampling variation, so these standard deviations directly indicate whether the chosen number of gene drops is adequate; no additional computation by the user is required. Monte Carlo sampling variation is also the only stochastic component of the estimates: identity-by-descent events are counted within each thread in exact 32-bit integer accumulators over independent replicate batches and combined only once at the end of the run, so integer overflow or floating-point round-off cannot occur even at 10⁹ replicates. The quality control based on sibling deviations in inbreeding coefficients, however, is not a direct measure for deviations in inbreeding coefficients for the entire pedigree across the simulations, since full siblings consist only a fraction of the total pedigree. Nevertheless, it is a straightforward and useful indicator of the adequate number of replicates for a given pedigree.

### Output and graphical summaries

PedXylo produces a comprehensive set of output files that support detailed assessment of pedigree structure and inbreeding patterns. Individual autosomal and X-chromosomal inbreeding coefficients, along with pedigree quality assessment metrics, are exported to separate text files, enabling their direct use in downstream analyses and hypothesis testing, such as studies of purging and inbreeding depression. At the population level, the software generates a CSV summary file containing descriptive statistics for all estimated parameters, including the number of individuals, mean, median, minimum, maximum, variance, standard deviation, 95% confidence interval, and 95% highest density interval. It also reports estimates of effective population size and pedigree quality metrics. In addition, PedXylo provides graphical summaries as PNG files, including histograms of inbreeding coefficients and pedigree symmetry scores, together with scatter plots illustrating the relationships between autosomal and X-chromosomal coefficients and between complete generation equivalent (CGE) [32] and inbreeding coefficients.

## New parameters and extensions implemented in PedXylo

### User-defined founder reference population

In most pedigree software, the founder reference population (FRP; Appendix Reference framework), the set of individuals against which identity-by-descent (IBD) is evaluated, is determined directly from the pedigree as the set of all individuals with unknown parents, which are assumed to be mutually unrelated and non-inbred. PedXylo retains this default but additionally allows the user to shift the pedigree base forward in time by specifying a birth year or a birth date threshold. When the threshold is specified, the immediately preceding individuals born before are automatically treated as founders: their recorded parental links are ignored, and each receives unique, mutually non-IBD alleles in the gene-dropping simulation, whereas individuals born in or after the threshold retain their recorded parents. Other individuals falling outside of FRP are deleted from the pedigree for subsequent analysis. The conversion is performed automatically by the program, and no manual editing of the pedigree file is required. Consequently, the FRP consists of pre-threshold individuals together with all later-born individuals with unknown parents; the ancestry of every analysed individual therefore terminates in FRP members, so that all coefficients remain computable, with the inbreeding of the redefined founders themselves being zero by construction. All reported parameters then quantify IBD accumulated exclusively through pedigree loops formed after the threshold.

Shifting the pedigree base in this controlled way is useful in several situations: in analyses targeting recent rather than distant inbreeding (for example, inbreeding accumulated over approximately the last five generations); when a known demographic shift in the past, as frequently documented in horse populations, would otherwise distort the estimation of effective population size, which should then be expressed relative to a historically meaningful baseline; when the earliest pedigree records are considered less reliable and would otherwise contaminate the estimates; in datasets where individuals with unknown parents reflect incomplete historical recording rather than true biological founders; in populations with uneven pedigree depth; and in populations for which pedigree registration began only after a certain period.

### Pedigree quality metrics: asymmetrical pedigree equivalent (APE) and pedigree tree imbalance (PTI)

Alongside standard pedigree-quality measures such as the complete generation equivalent [32], PedXylo introduces two new metrics that quantify structural balance: APE, newly proposed in this study, and PTI, which adapts classical tree-balance theory [33, 34] to pedigrees.

APE quantifies the difference in recorded ancestral depth between the paternal and maternal lines of an individual j. PedXylo computes APE in two complementary forms, each based on a different measure of pedigree depth.

CGE-based APE (cAPE) uses the complete generation equivalent and is defined as cAPE_j_ = CGE_paternal−j_ – CGE_maternal−j_, where CGE_paternal_ and CGE_maternal_ are the complete generation equivalents of the father and mother, respectively. cAPE captures the difference in information content between the two parental lines, weighting all known ancestors by the number of generations separating them from individual j.

Maximum-generations-based APE (mAPE) uses the maximum number of recorded generations and is defined as mAPE_j_ = MaxG_paternal−j_ – MaxG_maternal−j_, where MaxG_paternal_ and MaxG_maternal_ are the maximum topological generations represented in the paternal and maternal pedigrees of individual j, respectively. mAPE captures the difference in the deepest reach of each parental line, regardless of how completely the intervening generations are recorded.

For both forms, a value of zero indicates a balanced pedigree with equal depth across both parental lines; positive values indicate greater paternal-line depth and negative values greater maternal-line depth. APE captures recording asymmetry, which may arise from the introduction of individuals from unknown populations or from systematic biases in pedigree registration. APE is informative as a signed quantity; in some applications its absolute value (|cAPE| or |mAPE|) may also be used.

PTI quantifies the topological asymmetry of a pedigree by treating it as a directed acyclic graph approximating a rooted binary tree. Following Sackin [33] and Shao and Sokal [34], PTI is defined as PTI = d̄ / log₂(N_leaves_), where d̄ is the mean topological depth of the non-breeding individuals (leaves) in the pedigree (i.e. the mean MaxG across all non-breeding individuals) and N_leaves_ is the number of non-breeding individuals represented in the pedigree. The ratio is analogous to the normalised Sackin index of a binary tree [35]. Stray leaves, i.e. non-breeding individuals with no recorded ancestral depth, are excluded from the calculation; their number is reported separately by the program. For a perfectly balanced binary tree, the number of leaves and the depth are related by N_leaves_ = 2^d̄^, so that d̄ = log₂(N_leaves_) and PTI = 1.0. Departures from PTI = 1.0 indicate increasing asymmetry in the distribution of ancestral contributions across lineages and generations, with larger deviations corresponding to greater pedigree skewness.

Both APE and PTI reflect recording asymmetry, which may arise from the introduction of individuals from unknown populations or from systematic biases in pedigree registration. These two sources have opposite interpretations: genuinely unrelated immigrants truly reset inbreeding in their descendants, whereas unrecorded relationships caused by registration bias hide existing inbreeding and relatedness, biasing inbreeding estimates and, consequently, the rate of inbreeding and the effective population size. Like majority of pedigree-based software, PedXylo assumes that individuals with unknown parents are unrelated, non-inbred founders and cannot distinguish between these two scenarios from pedigree data alone. The role of APE and PTI is therefore diagnostic rather than corrective: divergent values flag individuals or population segments in which the estimates depend strongly on the founder assumption, so that they can be inspected, re-analysed with a redefined founder reference population (Sect.  User-defined founder reference population), or excluded from summary parameters such as the effective population size, while a definitive distinction between the two scenarios requires additional information (e.g., genomic data).

### Sex-linked inbreeding coefficients and their partial decomposition

Because of the sex-specific transmission of the X chromosome, X-linked inbreeding follows genealogical rules different from those of autosomal inheritance: males are hemizygous and cannot be inbred at X-linked loci, and fathers transmit their single X chromosome exclusively to daughters, so X-linked identity-by-descent depends on the sequence of sexes along each pedigree path (see Appendix Sex-linked inbreeding coefficients for a full account) [24]. PedXylo implements a sex-linked counterpart for each autosomal inbreeding coefficient (F_X_, F_NX_, F_aBX_, F_aKX_, and AHC_X_; Appendix Sex-linked inbreeding coefficients for a full account).

Together with their autosomal counterparts (F, F_N_, F_aB_, F_aK_, AHC), these five coefficient families are computed both at the whole-pedigree level and as ancestor-resolved (partial) decompositions, yielding a comprehensive partial-coefficient framework that, to our knowledge, is not currently available in any other pedigree-analysis tool. The implementation builds on the gene-dropping framework introduced in GRain [21] and extends it to X-linked inheritance and to integrated pedigree-quality assessment. Other software supporting partial coefficients, such as ENDOG [14], PEDIG [20], PyPedal [18], and PARTINBR [36], implements a more limited subset of these decompositions, generally for autosomal inheritance only.

### Per-individual rate of inbreeding (ΔF and ΔF_X_)

PedXylo computes the per-individual rate of inbreeding for every animal as a direct output, following the depth-corrected formulation of Gutiérrez et al. [27], ΔF_j_=1−(1 − F_j_)^1/(CGE^_j_^−1)^, where CGE_j_ is the complete generations equivalent for individual j. ΔF calculated in this way corrects classical F for pedigree depth and has been suggested to detect inbreeding depression with greater sensitivity than F itself [26]. PedXylo additionally computes the X-linked counterpart ΔF_X_ using F_X_ in place of F in the same formula. To our knowledge, no existing pedigree-analysis software produces ΔF_X_ as direct output.

### X-linked effective population size (Ne_X_)

PedXylo estimates X-linked effective population size as the size of an idealised population producing a rate of genetic drift, expressed as ΔF_X_, equal to that observed for X-linked loci in the study population. Ne_X_ is computed from the gene-dropping-based estimate of the X-linked rate of inbreeding and complements the autosomal realised effective population size [27] reported by PedXylo. Because Ne_X_ is sensitive to sex-specific inheritance, it can reveal unequal male and female contributions, sex-biased migration, and sex-specific variation in reproductive success that autosomal Ne does not capture [37, 38]. Under idealised assumptions, X-linked effective population size is expected to approximate three-quarters of autosomal Ne [39]; departures from this expectation in real pedigrees are themselves informative. Pedigree-based Ne_X_ estimates may be particularly useful as expectation-based comparators for genomic Ne_X_, which can also reflect recombination-rate variation, linked selection, and restricted introgression along the X chromosome [40]. Empirical validation of pedigree-based Ne_X_ against genomic estimates in pedigreed populations will be a useful direction for further work.

## Application examples

### Habsburg family pedigree

We used the Habsburg family, also presented in Álvarez et al. [1], as an illustrative example because this historically well-known pedigree, which includes prominent rulers such as Charles V, Philip II, Philip IV, and Charles II of Spain, is strongly shaped by consanguineous unions yet remains sufficiently compact for manual inspection. Comprising 37 individuals born between 1430 and 1661, it provides a multigenerational pedigree well suited to illustrating individual-based parameters and pedigree metrics, as readers can relate the estimated values to identifiable pedigree loops, ancestral contributions, and variation in pedigree structure among individuals. The presence of both male and female lineages, including siblings, also makes this pedigree useful for illustrating sex-linked inheritance, while differences in pedigree depth among individuals help demonstrate the consequences of incomplete pedigree information (Figs. 1 and 2).

### Autosomal and sex-linked inbreeding coefficients

Five terminal individuals born between 1605 and 1661 were selected to illustrate PedXylo-derived values for the main parameters and pedigree quality metrics (CGE_j_ and cAPE_j_), including autosomal and sex-linked inbreeding coefficients, as well as current (F_j_, F_Nj_, and ΔF_j_) and ancestral (F_aBj_, F_aKj_, and AHC_j_) inbreeding-related parameters (Table 1).

**Table 1:** Inbreeding and pedigree quality metrics for selected individuals from the Habsburg pedigree

	Individual (pedigree ID, short name & life span)	Sex	F_j_	F_Nj_	F_aBj_	F_aKj_	AHC_j_	CGE_j_	cAPE_j_	ΔF_j_
Autosomal inbreeding coefficients	33 Philip IV (1605-1665)	Male	0.0908	0.0560	0.1802	0.0348	0.1953	4.88	1.00	0.0242
	34 Maria Anna (1606-1646)	Female	0.0908	0.0560	0.1802	0.0348	0.1953	4.88	1.00	0.0242
	35 Ferdinand III (1608-1657)	Male	0.0000	0.0000	0.0625	0.0000	0.0625	2.68	3.38	0.0000
	36 Mariana (1634-1696)	Female	0.1001	0.0598	0.1571	0.0411	0.1743	4.78	-2.19	0.0275
	37 Charles II (1661-1700)	Male	0.2172	0.1064	0.2417	0.1108	0.2806	5.83	0.09	0.0494
		Sex	F_Xj_	F_NXj_	F_aBXj_	F_aKXj_	AHC_Xj_	CGE_j_	cAPE_j_	ΔF_Xj_
Sex-linked inbreeding coefficients	33 Philip IV (1605-1665)	Male	n.d.	n.d.	0.0625	0.0625	0.0000	4.88	1.00	n.d.
	34 Maria Anna (1606-1646)	Female	0.1778	0.1406	0.1094	0.0371	0.1094	4.88	1.00	0.0494
	35 Ferdinand III (1608-1657)	Male	n.d.	n.d.	0.0000	0.0000	0.0000	2.68	3.38	n.d.
	36 Mariana (1634-1696)	Female	0.0000	0.0000	0.1343	0.0000	0.1436	4.78	-2.19	0.0000
	37 Charles II (1661-1700)	Male	n.d.	n.d.	0.0671	0.0000	0.0718	5.83	0.09	n.d.

F is classical inbreeding coefficient; F_N_ is Kalinowski's new inbreeding coefficient; F_aB_ is Ballou's ancestral inbreeding coefficient; F_aK_ is Kalinowski's ancestral inbreeding coefficient; AHC is ancestral history coefficient; CGE is complete generation equivalent; cAPE is CGE-based asymmetrical pedigree equivalent (this study); ΔF is individual rate of inbreeding. In the sex-linked block, the subscript X denotes the X-linked counterpart of the corresponding coefficient; F_X_, F_NX_, and ΔF_X_ are not defined (n.d.) for hemizygous males. The coefficients reported here derive from the 37-individual pedigree, whereas Álvarez et al. [1] used a pedigree of ~3,000 individuals spanning 16 generations

The divergence between autosomal and sex-linked inheritance is strikingly illustrated by individual 36 (Mariana, 1634–1696). Although she exhibits a high level of autosomal inbreeding (F_j_ > 0.1000), all corresponding sex-linked coefficients (F_Xj_, F_NXj_, F_aKXj_, and ΔF_Xj_) were zero (Table 1). This discrepancy highlights a fundamental constraint of X-linked transmission: because a father does not transmit his X chromosome to his son, any pedigree loop (a closed path formed when the paternal and maternal lineages of an individual meet in a common ancestor) containing two successive males effectively interrupts the transmission path for X-linked autozygosity. In Mariana’s ancestry, such male-to-male transmissions occur sequentially from individual 23 (Charles II, 1540–1590) to individual 31 (Ferdinand II, 1578–1637), and then to individual 35 (Ferdinand III, 1608–1657), as indicated by blue arrows in Fig. 2. Interestingly, although her current sex-linked inbreeding is zero, Mariana exhibits non-zero values for ancestral sex-linked coefficients (F_aBXj_ and AHC_Xj_), although these remain lower than their autosomal counterparts (F_aBj_ and AHC_j_), reflecting historical autozygosity in her maternal lineages (Table 1).

Notably, standard sex-linked inbreeding coefficients (F_Xj_, F_NXj_, and ΔF_Xj_) are theoretically undefined for hemizygous males, such as those presented in Table 1, because autozygosity is a property of diploid loci. However, PedXylo implements a “relaxed” definition for the sex-linked ancestral coefficients (F_aBXj_, F_aKXj_, and AHC_Xj_). These metrics remain highly informative in males because they quantify the historical autozygosity of the specific X-chromosomal haplotype they inherited. By identifying the past exposure of X-linked alleles to a homozygous state in female ancestors, these coefficients provide a robust basis for testing hypotheses related to the purging of X-linked recessive deleterious alleles.

Differences are also clearly visible when comparing autosomal and sex-linked coefficients between individual 33 (Philip IV, 1605–1665) and his sister, individual 34 (Maria Anna, 1606–1646). Although these two individuals have identical values for all autosomal coefficients and pedigree quality metrics, markedly different values were obtained for the sex-linked coefficients (Table 1).

Considerable effort was invested in the inclusion and development of pedigree quality assessment metrics, which constitute an important feature and strength of PedXylo. Previous studies have shown that pedigree quality is essential for the reliable interpretation of many inbreeding coefficients calculated in PedXylo [41, 42], as their definitions inherently make them strongly dependent on pedigree completeness and depth. Even in this small example, this dependency is clearly evident: individual 37 (Charles II, 1661–1700), who exhibited the highest level of inbreeding, also had the highest CGE, whereas individual 35 (Ferdinand III, 1608–1657), for whom F_j_, F_Nj_, F_aKj_, and ΔF_j_ were all equal to zero, had the lowest CGE (Table 1). At the same time, the markedly outlying cAPE value observed for individual 35 highlights the extremely limited pedigree information available for this individual, caused by the inclusion of his mother, Maria Anna (1574–1616), without genealogical background (Table 1; Fig. 2). This absence of maternal ancestral information also introduced asymmetry in pedigree completeness. Overall, PTI for this pedigree is 6.00 with only 2 leaves of depths 4 and 8 (Charles and Charles II respectively), which constitutes a large deviation from 1.00 for perfectly balanced pedigrees, a feature also visible in Fig. 2. While such reduced pedigree informativeness may still be visually recognized in small pedigrees, pedigree asymmetry metrics are likely to be especially valuable in large pedigrees, where visual inspection becomes impossible.

### Partial inbreeding coefficients

Except for ΔF_j_ and ΔF_Xj_, all other individual autosomal and sex-linked inbreeding coefficients calculated in PedXylo can be decomposed into partial inbreeding coefficients attributable to specific founders or ancestors.

This feature makes PedXylo the most comprehensive tool currently available for calculating partial inbreeding coefficients. Here, the concept of partial inbreeding coefficients is illustrated for individual 34 (Maria Anna, 1606–1646) from the Habsburg pedigree with respect to 10 founders (Table 2). The sum across all 10 founders was equal to the corresponding non-partial coefficients, with only minor discrepancies caused by rounding. This demonstrates that the concept of partial coefficients provides a valid decomposition of individual inbreeding coefficients; compare the total sum of partial coefficients in Table 2 with the values reported in Table 1 for individual 34 (Maria Anna, 1606–1646). Table 2 shows that founder contributions varied depending on the coefficient analysed. For example, individuals 3, 4, 7, and 8 contributed to all partial coefficients calculated by PedXylo for individual 34. In contrast, individuals 25 and 32 did not contribute to any of the inbreeding coefficients, whereas individuals 5 and 6 contributed only to the autosomal inbreeding coefficients, as X-chromosome transmission was interrupted by two consecutive male-to-male gametic segregations within their inbreeding loop (Fig. 2).

**Table 2:** Partial ancestral inbreeding coefficients for Maria Anna of Spain (1606–1646)

*Founder / Partial Coefficient	F_j_	F_Nj_	F_aBj_	F_aKj_	AHC_j_	F_Xj_	F_NXj_	F_aBXj_	F_aKXj_	AHC_Xj_
1 Ferdinand (1433–1470)	0.0000	0.0000	0.0078	0.0000	0.0078	0.0000	0.0000	0.0000	0.0000	0.0000
2 Beatriz (1430–1506)	0.0000	0.0000	0.0078	0.0000	0.0078	0.0000	0.0000	0.0000	0.0000	0.0000
3 Ferdinand (1452–1516)	0.0190	0.0094	0.0442	0.0097	0.0508	0.0117	0.0078	0.0313	0.0039	0.0313
4 Elizabeth (1451–1504)	0.0191	0.0094	0.0442	0.0097	0.0508	0.0098	0.0078	0.0156	0.0020	0.0156
5 Maximilian I (1459–1519)	0.0146	0.0089	0.0225	0.0058	0.0234	0.0000	0.0000	0.0000	0.0000	0.0000
6 Mary (1457–1482)	0.0147	0.0089	0.0225	0.0058	0.0234	0.0000	0.0000	0.0000	0.0000	0.0000
7 Vladislaus II (1456–1516)	0.0117	0.0098	0.0156	0.0020	0.0156	0.0781	0.0625	0.0313	0.0156	0.0312
8 Anne (1484–1506)	0.0117	0.0098	0.0156	0.0020	0.0156	0.0781	0.0625	0.0312	0.0156	0.0312
25 Albert V (1528–1579)	0.0000	0.0000	0.0000	0.0000	0.0000	0.0000	0.0000	0.0000	0.0000	0.0000
32 Maria Anna (1574–1616)	0.0000	0.0000	0.0000	0.0000	0.0000	0.0000	0.0000	0.0000	0.0000	0.0000
Summation of all partial coefficients	0.0908	0.0562	0.1802	0.0350	0.1952	0.1777	0.1406	0.1094	0.0371	0.1093

*Founders are presented with pedigree ID, short name life span (birth year – death year)

All results for this example were obtained with a single command of the command-line version, *xylo_cl --pedigree Habsburg_ped_37i.csv --nsim 1,000,000,000 --nf 10 --n-thread 8 --sx-chrom true*, which performs pedigree checking, gene dropping under both inheritance modes, and exports of all tabular and graphical outputs; the same analysis can be performed interactively in the GUI version.

### European bison pedigree

The pedigree of the European bison population from Białowieża National Park, described by Machová et al. [43], was used to illustrate the application of PedXylo in population-level pedigree analysis. This pedigree was selected because it is deep, essentially complete, and publicly available, which allows full reproducibility of the presented analyses. The pedigree includes individuals born between 1881 and 2020, and the results presented here refer to a target reference population (TRP) of 1807 bison alive on 31 December 2019. This TRP differs slightly from the one defined by Machová et al. [43]. For individuals with unrecorded sex, PedXylo infers sex from the pedigree where possible, assigning sex to those that appear as a parent (sire or dam) of recorded offspring. Sex remains undefined only for individuals with unrecorded sex and no offspring, and sex-linked inbreeding coefficients are not computed for these individuals; this approach ensures that results are fully reproducible across runs. Three offspring-less individuals of unknown sex were assigned a sex in the example pedigree to simplify presentation of the results.

The file ped_Bison_run.log provides details on program execution as well as pedigree characteristics. In this example, analyses were performed on a UNIX platform for both autosomal and X-chromosomal gene dropping, using 10,000,000 simulations (drops) across 40 threads; total runtime was 5.8 min. The analysed pedigree comprised 15,146 individuals, including 14,669 original animals and 477 additional founders generated by PedXylo from half-orphan records. The inclusion of these additional founders was necessary because gene-dropping analysis assumes that each founder has two unknown parents and contributes unique alleles to the segregation process; therefore, half-orphan founders could not be accommodated without the automatic adjustment implemented in PedXylo.

Females were slightly more numerous than males across the entire analysed pedigree of 15,146 individuals, with a male-to-female ratio of 0.96. Across the entire pedigree, the mean CGE was 6.70, the mean cAPE was 1.35, and PTI was 1.13. As reported in the ped_Bison_generation_interval.txt file, the mean APO was 8.95 years, averaged over all four parental pathways: sire–son (8.95), sire–daughter (8.94), dam–son (8.92), and dam–daughter (8.96).

Table 3 presents summary statistics for all individual-level parameters calculated by PedXylo for the target reference population of 1807 European bison alive on 31 December 2019, including 709 males and 1098 females (male-to-female ratio 0.65). Because the X-linked parameters F_X_, F_NX_, and ΔF_X_ are not defined for hemizygous males, their summary statistics are based on the 1098 females, whereas all other parameters were summarised using the full set of 1807 individuals.

**Table 3:** Summary statistics of the Białowieża National Park bison pedigree

Parameter	Mean	Median	Range	Variance
F	0.17082	0.14037	0.00000–0.71838	0.01921
F_X_	0.23645	0.20058	0.00000–0.87678	0.03947
F_N_	0.01701	0.00636	0.00000–0.25015	0.00082
F_NX_	0.02378	0.00000	0.00000–0.50003	0.00292
ΔF	0.02298	0.01656	0.00000–0.68384	0.00075
ΔF_X_	0.03417	0.02476	0.00000–0.24994	0.00133
F_aB_	0.59524	0.65238	0.00000–0.96445	0.04528
F_aBX_	0.48956	0.47137	0.00000–0.96604	0.06553
F_aK_	0.15381	0.12926	0.00000–0.70450	0.01653
F_aKX_	0.12923	0.00746	0.00000–0.82613	0.03055
AHC	1.61840	1.56849	0.00000–5.11847	0.66774
AHC_X_	1.16418	1.10465	0.00000–4.21117	0.53635
CGE	8.98	9.94	0.00–13.40	9.53
cAPE	0.63	0.00	–11.14–11.37	9.90

F is classical inbreeding coefficient; F_N_ is Kalinowski’s new inbreeding coefficient; ΔF is individual rate of inbreeding; F_aB_ is Ballou’s ancestral inbreeding coefficient; F_aK_ is Kalinowski’s ancestral inbreeding coefficient; AHC is ancestral history coefficient; CGE is complete generation equivalent; cAPE is CGE-based asymmetrical pedigree equivalent (this study). The subscript X (F_X_, F_NX_, ΔF_X_, F_aBX_, F_aKX_, AHC_X_) denotes the X-linked (sex-linked) counterpart of the corresponding autosomal parameter

Researchers can obtain summary statistics for pedigree-based genetic parameters either from the .txt output files generated by the program, ped_Bison_tot.txt for autosomal results and ped_Bison_X_tot.txt for sex-linked results, or directly from the summary output file, ped_Bison_Summary_statistics.txt. Likewise, pedigree quality assessment metrics can be retrieved from the corresponding output file, here ped_Bison_CGE_DCE_values.txt. In addition to tabular outputs, PedXylo also generates graphical presentations (.png files) that illustrate the distributions of pedigree-based genetic parameters and the relationships among them.

**Fig. 3:**
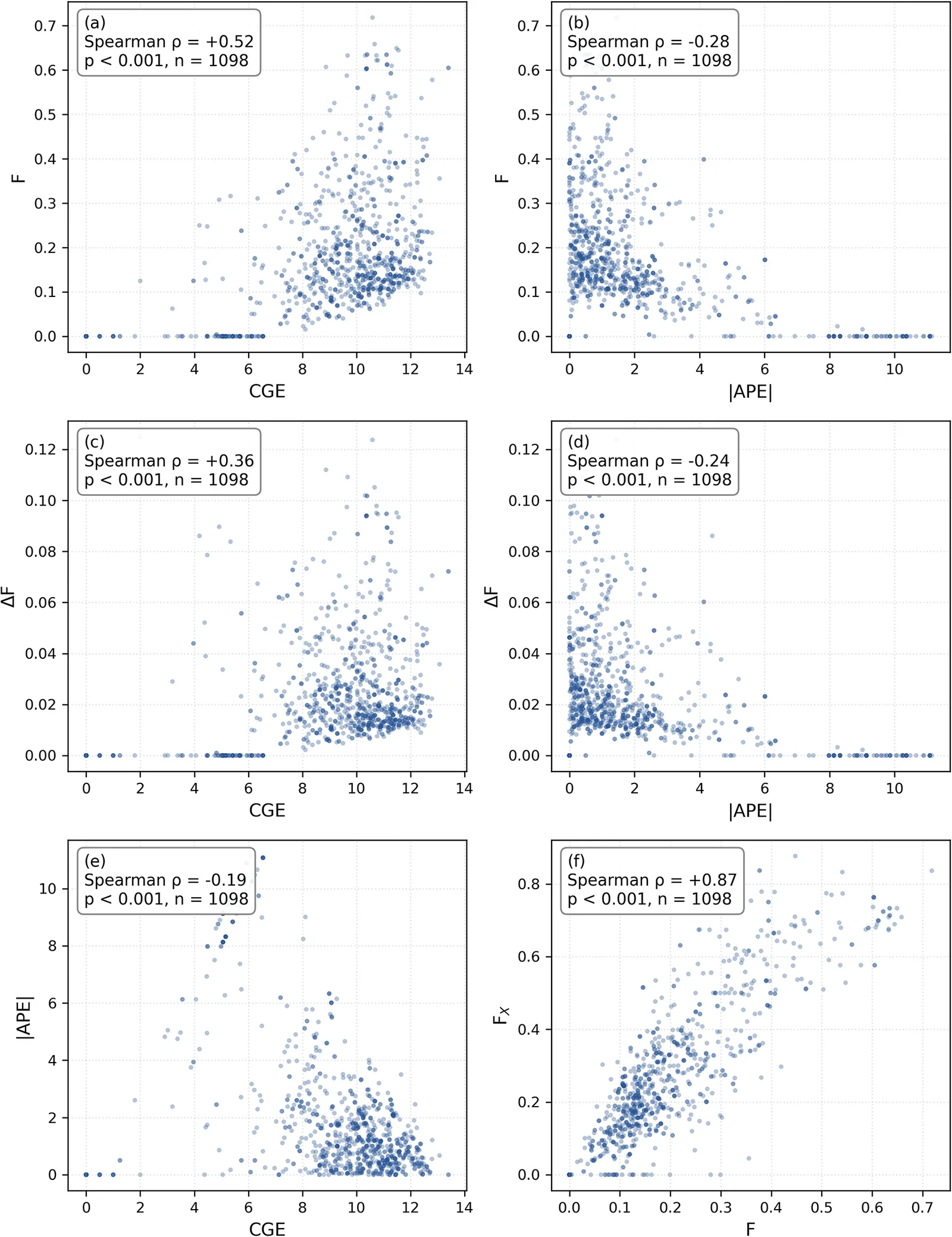
Bison scatter plots. Scatter plots illustrating the relationships among selected individual-level parameters calculated in PedXylo: (**a**) F and CGE, (**b**) F and |APE|, (**c**) ΔF and CGE, (**d**) ΔF and |APE|, (**e**) CGE and |APE|, and (**f**) F and F_X_. All panels are based on the 1098 females of the target reference population (n = 1098), since F_X_ (panel f) is defined only for females; the same set was used in all panels for comparability

Figure 3 presents the relationships among F, ΔF, CGE, |APE|, and F_X_ for the female reference population. These comparisons are particularly relevant because pedigree-based genetic parameters, in particular inbreeding coefficients, are strongly influenced by the depth, completeness, and structural asymmetry of the pedigree. The effects of pedigree information content (CGE) and pedigree asymmetry, expressed as the absolute value of APE (|APE|), on the inbreeding coefficient are shown in Fig. 3a, b. Both pedigree-quality metrics were substantially associated with variation in F (Spearman ρ = 0.52 for CGE and − 0.28 for |cAPE|). By contrast, the weaker correlation of ΔF with CGE (ρ = 0.36, versus 0.52 for F; Fig. 3c, d) is consistent with the pedigree-depth correction built into ΔF, whereas its correlation with |cAPE| remained essentially unchanged (− 0.24, versus − 0.28 for F), indicating that the depth correction does not remove the association with pedigree asymmetry. These correlations are descriptive for this population and are presented to illustrate the diagnostic outputs of PedXylo rather than to establish general relationships among the parameters.

The weak correlation between CGE and |cAPE| (ρ = −0.19; Fig. 3e) indicates that pedigree asymmetry captures an aspect of pedigree quality that is at least partly independent of pedigree depth and completeness and may therefore contribute additional explanatory power when interpreting variation in inbreeding-related coefficients.

Finally, Fig. 3f illustrates the relationship between autosomal and sex-linked inbreeding coefficients, a topic that has received limited attention but can now be readily evaluated. In the European bison pedigree, the two coefficients were strongly correlated (Spearman ρ = 0.87), although this pattern was not consistently observed across other populations analysed in our evaluation of PedXylo.

In this study, we present selected examples of the graphical output generated by PedXylo as .png files, whereas the complete set of PedXylo-derived analyses assessing the influence of pedigree information on variation in pedigree-based genetic parameters, as well as the relationships among these coefficients, is provided in the Supplementary Material (Bison_all_results_ref-pop.zip). Because relationships involving ΔF were not available directly in the graphical output, they were derived separately from the corresponding .txt output files.

Although PedXylo provides individual increases in inbreeding for autosomal and sex-linked inheritance (ΔF and ΔF_X_), which can be used to estimate effective population size, these values are also reported in the corresponding .txt output file (for the European bison TRP, ped_Bison_Effective_population.txt). In the European bison TRP analysed here, Ne was 21.8 and Ne_X_ was 14.6, giving an Ne_X_/Ne ratio of 0.67, somewhat below the theoretical expectation of 0.75 under standard assumptions for X-linked relative to autosomal effective population size [44].

Overall, in the TRP of our European bison pedigree, sex-linked inbreeding coefficients were higher than the corresponding autosomal coefficients (F_Xj_ > F_j_, F_NXj_ > F_Nj_, and ΔF_Xj_ > ΔF_j_), whereas the opposite pattern was observed for ancestral coefficients, all of which were higher for autosomal than for sex-linked estimates (F_aBj_ > F_aBXj_, F_aKj_ > F_aKXj_, and AHC_j_ > AHC_Xj_) see Table 3.

In the European bison pedigree, 368 cases with two or more siblings were used to evaluate the precision of the estimated coefficients obtained from 10^7^ gene-dropping runs (see ped_Bison_sibling_QC.txt). The mean standard deviations were 0.00008 for F_j_, 0.00002 for F_Nj_, 0.00006 for F_aBj_, 0.00007 for F_aKj_, and 0.00020 for AHC_j_. Because ΔF_j_ is a deterministic transformation of F_j_ and CGE_j_, and CGE carries no Monte Carlo error, the precision of ΔF_j_ follows directly from that of F_j_ [SD(ΔF) ≈ SD(F)/(CGE − 1)], i.e., approximately 0.00001 in this pedigree); the same holds for ΔF_Xj_ with respect to F_Xj_. This indicates that the estimates were already highly precise, and an even greater level of precision would be expected with a larger number of gene-dropping runs.

The corresponding command for this example was *xylo_cl --pedigree ped_Bison.csv --nsim 10,000,000 --nout 1 --n-thread 40 --sx-chrom true --trp ref_Bison.txt*; the run log distributed with the software (ped_Bison_run.log) documents the complete execution.

We have also tested the performance of PedXylo on this pedigree against GRain2.2 using only 1 CPU core on a computer system with Intel Core i7-4770 @ 3.40 GHz, 31 GB RAM, Pop! _OS 22.04 and at 10^7^ iterations only for autosomal inbreeding we obtained the following runtimes: GRain 245.08 min and PedXylo 53.8 min. This represents a significant improvement even without multithreading included.

## Computational performance on industry-scale

To assess the scalability of PedXylo to pedigree sizes encountered in national-scale animal breeding, we benchmarked the program on the Czech Holstein dairy-cattle pedigree of 23,243,118 individuals.

**Fig. 4:**
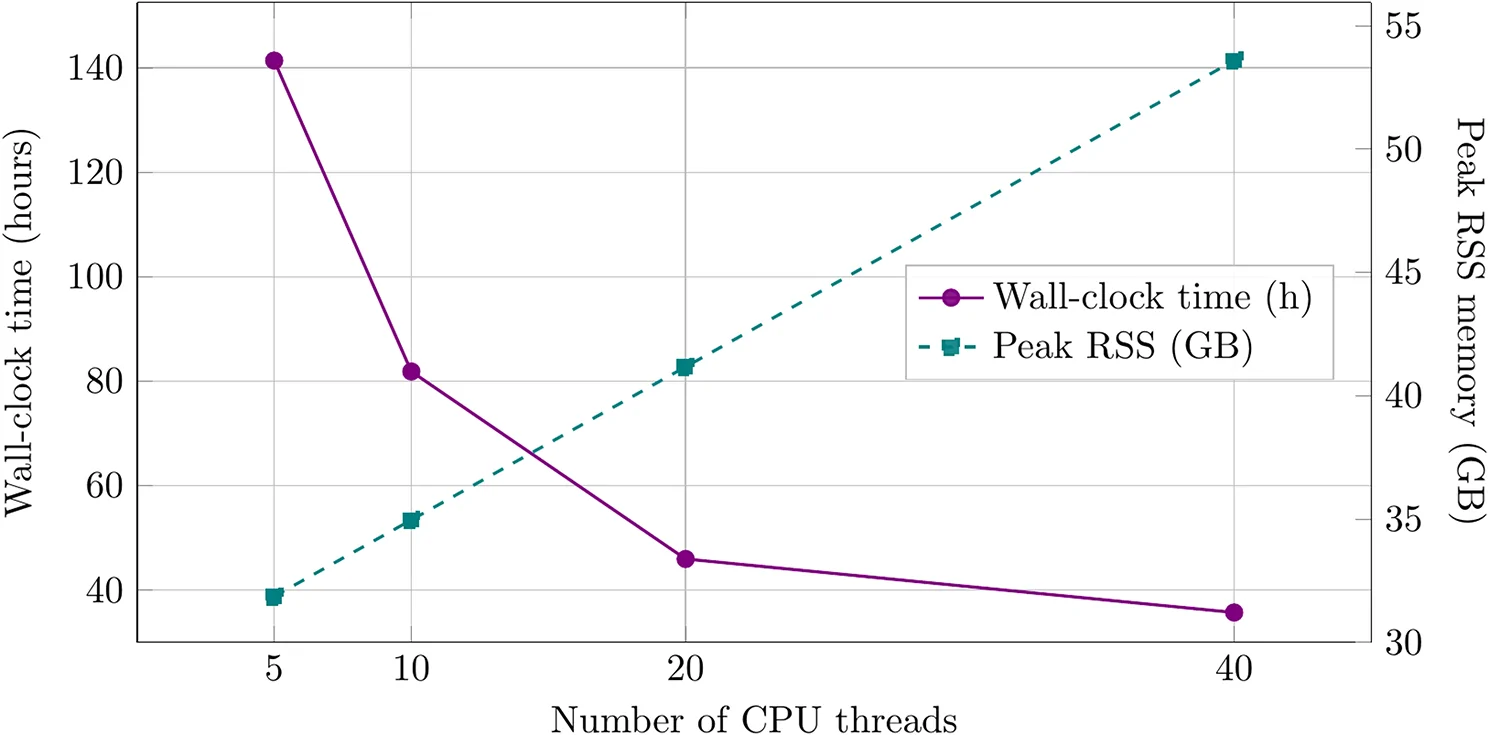
Computational requirements. Wall-clock times (in hours) and peak resident set size (RSS) memory requirements for 1 million iteration long gene-dropping simulations performed on Czech Holstein dairy cattle pedigree (size = 23,243,118) in dependence of the number of allocated CPU threads

Automated pre-processing imputed 18 undefined ancestors and extended 10 half-orphan records, yielding 23,243,146 total records. All analyses were run on a single workstation (Intel Xeon Silver 4316, 40 threads; 512 GB RAM; Fedora Linux 43). Following Baumung et al. [21], all gene-dropping runs used 10⁶ replicates. Pedigree reading took 10.2 s, pedigree integrity checking and population analysis required approximately 7.2 min. Autosomal and X-linked gene-dropping completed in approximately 71 h, 41 h, 23 h and 18 h for each inheritance regime, using 5, 10, 20 and 40 threads as presented in Fig. 4. Posterior generation of textual and graphical outputs required additional 6.9 min. Peak memory consumption was approximately 32, 35, 41 and 53 GB respectively, well below the 512 GB available, indicating that PedXylo can be run on standard high-memory workstations rather than requiring distributed-computing infrastructure.

As Fig. 4 shows, the benefit of allocating additional CPU threads diminishes progressively, as inter-thread communication overhead comes to dominate. In this case the practical limit is set by the hardware: the Intel Xeon Silver 4316 processor provides 20 physical cores and 40 logical threads through simultaneous multithreading (SMT), so allocating all 40 threads added limited speed-up beyond what 20 threads already achieved.

In most practical scenarios, the program is expected to be applied to pedigrees similar in size to the European bison pedigree analysed in this study, for which approximately 5.8 min were required to perform 10^7^ gene-dropping runs on 40 CPU threads.

## Discussion

### PedXylo in the landscape of pedigree software

PedXylo is intended to complement rather than replace existing pedigree-analysis software. For analyses centred on the construction of relatedness matrices for animal-model estimation of additive, dominance, and epistatic variances, the nadiv R package [17] remains more appropriate. For pedigree-based simulation of genetic management in small captive populations, including the evaluation of breeding circles, mating restrictions, and outcrossing scenarios, Retriever and Pointer [19] and PMx [8] offer dedicated frameworks, while Vortex [45] provides a platform for inbreeding depression simulations and population viability analysis. For the routine handling of very large pedigrees in command-line workflows, RelaX2 [15] and Pedig [20] remain efficient and well-suited tools. Within this landscape, PedXylo offers a broad set of routine pedigree analyses. These include pedigree consistency checking and correction, inbreeding-level monitoring, founder-resolved analysis of inbreeding-depression heterogeneity, the study of purging of deleterious recessive variants through ancestral coefficients, and the estimation of effective population size. Beyond these standard capabilities, PedXylo introduces the integration of autosomal and X-linked inbreeding analyses and a comprehensive partial-coefficient framework spanning all five inbreeding-coefficient families on both autosomes and the X chromosome. It also implements the new pedigree-quality metrics APE and PTI, provides direct estimation of X-linked effective population size, and has demonstrated scalability to multi-million-individual pedigrees. PedXylo therefore broadens the pedigree-analysis toolbox in directions that complement rather than duplicate the strengths of existing software.

### Biological relevance of sex-linked pedigree analysis

The application examples presented in this study demonstrate that sex-linked coefficients can reveal biologically informative patterns that remain invisible when analyses are restricted to autosomal metrics. In the Habsburg family pedigree, the case of Mariana (individual 36) illustrates how pedigree loops containing consecutive male-to-male transmissions interrupt X-linked identity-by-descent, producing zero sex-linked inbreeding despite substantial autosomal inbreeding. Conversely, the full-sibling pair of Philip IV (individual 33) and Maria Anna (individual 34) exhibited identical autosomal coefficients but markedly different sex-linked values, reflecting the sex-specific rules of X-chromosome transmission. These patterns are not artifacts; they have direct implications for understanding X-linked genetic load, the potential for sex-specific purging, and the interpretation of fitness differences between individuals with similar autosomal inbreeding levels. A concrete example of an X-linked gene with major phenotypic effects illustrates these implications: the BMP15 (FecX) locus in sheep increases ovulation rate in heterozygous ewes but causes sterility in homozygous carriers [46]. For such loci, X-linked autozygosity quantified by F_X_ and its ancestral counterparts directly determines the probability that recessive alleles are expressed in females, whereas hemizygous males express them regardless, and in flocks segregating FecX alleles, X-linked inbreeding therefore directly predicts the risk of producing sterile daughters.

At the population level, the European bison pedigree analysis revealed that sex-linked inbreeding coefficients were consistently higher than their autosomal counterparts, whereas ancestral coefficients showed the opposite pattern. The Ne_X_/Ne ratio of 0.67 fell below the theoretical expectation of 0.75, suggesting unequal sex-specific contributions, sex-biased demographic processes, or both. These findings underscore the value of jointly analysing autosomal and sex-linked parameters and demonstrate that the tools provided by PedXylo can generate testable hypotheses about mating systems and demographic history.

### The role of pedigree quality assessment

The strong dependence of inbreeding coefficients on pedigree depth and completeness is well documented (e.g. [47]), , yet in practice pedigree quality is often reported only as a mean CGE. Our results in the European bison reference population show that the two new metrics introduced here, cAPE and mAPE, are only weakly correlated with CGE (Spearman ρ = −0.07 and − 0.13, respectively), confirming that they capture aspects of pedigree quality not represented by depth alone. Both were also moderately correlated with the classical inbreeding coefficient F (ρ = −0.31 for |cAPE|, − 0.12 for |mAPE|), indicating that structural pedigree imbalance is associated with variation in F estimates. This suggests that reporting pedigree completeness alone may be insufficient, and that measures of structural balance should be considered when interpreting pedigree-based genetic parameters, particularly in populations with heterogeneous recording history. APE and PTI are likely to be most valuable in large pedigrees, where the scale of the data makes case-by-case visual inspection infeasible and quantitative summaries are required. It should be emphasised that APE and PTI are proposed as diagnostic metrics for assessing the reliability of pedigree-based estimates and for screening the recording history of a population, not as normalisation or correction factors.

### Limitations and considerations

Several limitations of the current version of PedXylo should be acknowledged. First, as a gene-dropping-based approach, all estimates are subject to stochastic sampling variation. Although 10^7^ gene-dropping runs yielded highly precise estimates in the examples presented here (mean standard deviation of F_j_ among full siblings = 0.00008), users working with extremely large pedigrees should evaluate the trade-off between precision and computational cost. Second, PedXylo does not currently compute pedigree-based relationship (kinship) matrices, which are required for certain applications in quantitative genetics and mixed-model analysis [48–50]. Third, the software assumes that the pedigree is correct as provided and does not incorporate marker-based parentage verification or pedigree reconstruction [51, 52]. In practice, pedigree errors can substantially bias inbreeding estimates [53], and users should verify pedigree accuracy before analysis. Fourth, while PedXylo detects basic structural errors such as sex inconsistencies and ancestry loops, it does not currently implement more advanced pedigree diagnostics such as the detection of implausible generation intervals or age–pedigree conflicts. Finally, PedXylo operates within the framework of classical pedigree-based IBD and does not model the stochastic variation in realized genomic IBD that arises from Mendelian sampling at finite genomes [54], neither does it model the allele selection events that lead to transmission ratio distortions such as gene purging, segregation distortion or gamete selection [55].

The future development of PedXylo will focus on two concrete objectives: the implementation of pedigree-based kinship matrices, including their X-linked counterparts; and the integration of marker-based parentage verification to identify pedigree errors before analysis.

### Practical recommendations

In practice, the choice of which parameters to compute and report depends on the question being asked. For describing the current inbreeding state of a population - for example, when assessing the immediate consequences of recent management decisions - the classical inbreeding coefficient F and its X-linked counterpart F_X_ are the primary quantities, ideally reported alongside ΔF and ΔF_X_ to control for variation in pedigree depth across individuals. For assessing historical inbreeding load - for example, when interpreting inbreeding depression in the context of possible past purging - the ancestral coefficients F_aB_, F_aK_, and AHC, and their X-linked counterparts, provide complementary perspectives on the inbreeding history of currently autozygous alleles. For investigating whether specific founders or ancestors contribute disproportionately to inbreeding depression, the partial-coefficient decompositions of any of these quantities allow inbreeding to be attributed to individual ancestors. For describing the demographic state of a population, autosomal Ne summarises the rate at which inbreeding is accumulating; Ne_X_ complements this by capturing sex-specific dynamics that autosomal Ne does not see.

Throughout, the reliability of any of these estimates depends on the quality of the underlying pedigree. Reporting CGE alongside any inbreeding statistic, and reporting APE and PTI when comparing individuals or sub-populations, is recommended. PedXylo produces these pedigree-quality metrics as standard output specifically to support transparent reporting of the basis on which inbreeding estimates are computed.

## Conclusions

PedXylo makes the complete family of pedigree-based inbreeding analyses available for the X chromosome and integrates them with pedigree management and quality assessment in a single free, open-source program. The application examples support three main conclusions. First, autosomal and X-linked inbreeding can diverge markedly at the individual level, as shown in the Habsburg pedigree, where individuals with high autosomal inbreeding exhibited zero X-linked inbreeding and full siblings differed only in their sex-linked values, and at the population level, as shown in the European bison, where X-linked coefficients exceeded their autosomal counterparts and the Ne_X_/Ne ratio of 0.67 fell below the theoretical expectation of 0.75, so that analyses restricted to autosomal parameters may hide biologically relevant variation in genetic load, purging opportunities, and demographic history. Second, pedigree depth alone does not capture pedigree quality: the structural-balance metric cAPE shows almost no monotonic association with CGE, and it flags individuals whose inbreeding estimates are potentially unreliable owing to one-sided recording. Third, the demonstrated scalability to a national pedigree of 23.2 million individuals on a single workstation shows that these analyses are feasible in routine animal breeding, as well as in conservation, zoo management, and human-isolate research.

## Data Availability

The source code, data, user instructions and preinstalled executables of PedXylo are available in a git repository https://www.gitlab.com/dhrsak/pedxylo.git. The pedigrees of the Habsburg family and the European bison population, together with the corresponding output files from the analyses presented here, are provided as supplementary material. The Czech Holstein cattle pedigree cannot be made publicly available because it contains data owned by individual farmers.

## Appendix 1

### Pedigree-based parameters: an introductory primer with formal definitions

#### A.1 Introduction

This appendix provides an introductory primer on the parameters computed by PedXylo, intended for readers new to pedigree analysis or seeking a consolidated reference. The parameters are organised into seven thematic groups: the reference framework that fixes what a pedigree analysis is computed against (A.2); pedigree-quality metrics that describe the structural reliability of the input data (A.3); classical autosomal inbreeding coefficients (A.4); ancestral inbreeding coefficients that summarise the inbreeding history of an individual’s alleles (A.5); partial inbreeding coefficients that decompose inbreeding by ancestor (A.6); the sex-linked counterparts of all the above (A.7); and effective population size (A.8). Each group opens with a short framing paragraph that explains what the parameters in that group capture and how they relate to one another, followed by formal definitions and the references in which the parameters were originally introduced.

#### A.2 Reference framework

Pedigree-based parameters are not absolute properties of an individual. They are evaluated relative to a defined reference framework consisting of the population whose properties are being estimated (the target reference population), the set of founders against which identity-by-descent (IBD) is assessed (the founder reference population), and the temporal scale on which generations are translated to chronological time (the generation interval). Two pedigree analyses that report different inbreeding levels for the same individual may simply be using different reference frameworks; clarifying these choices is therefore essential to interpreting any pedigree-based estimate.

##### Target reference population (TRP)

TRP is a defined group of individuals for which pedigree-based parameters are estimated and interpreted.

##### **Founder reference population (FRP).**

FRP is the set of founder individuals with unknown parents that defines the pedigree base, with all such individuals assumed to be mutually unrelated. By convention, alleles inherited from different founders are treated as non-IBD; consequently, estimates of pedigree-based parameters depend strongly on how the FRP is defined. PedXylo allows the FRP to be defined either as the default set of all individuals with unknown parents or explicitly using a birth-year threshold so that only immediate ancestors of individuals born after the specified year, as well as individuals with unknown parents born on and after the specified year are assigned to the FRP.

##### Generation interval (GI)

GI is classically defined as the average age of parents at the birth of their offspring that are themselves used for reproduction [31]. PedXylo computes and reports it across the four standard reproductive pathways (father–son, father–daughter, mother–son, mother–daughter) based only on offspring that appear as parents in the pedigree. In addition, PedXylo calculates and reports ages of parents at birth of offspring (APO) based on all recorded births across the four reproductive pathways as well. The comparison of GI and APO is informative about population management: GI is expected to exceed APO under conservation-type management and to fall below it under selection. Because recent cohorts may not yet have completed their reproductive careers, GI should be interpreted for cohorts with completed reproduction. GI links pedigree structure (generations) to chronological time (years) and is essential for translating per-generation parameters, such as the rate of inbreeding, into per-year quantities.

#### A.3 Pedigree quality metrics.

The reliability of any pedigree-based estimate depends on how much pedigree information is available and how that information is distributed across the genealogy. This section defines the metrics PedXylo computes to quantify pedigree depth (CGE, MaxG, NKA), generational completeness (GPC), and structural balance between the paternal and maternal sides of the pedigree (APE in two forms, PTI).

##### Complete generation equivalent (CGE)

CGE_j_ [32] is defined as the sum over all known ancestors of individual j of the term (1/2)^n^, where n is the number of generations separating individual j from each ancestor. CGE represents the number of complete generations that would provide the same amount of pedigree information.

##### Maximum number of generations (MaxG)

MaxG_j_ is the greatest number of successive ancestral generations recorded in the pedigree of individual j, following any lineage back to the most remote identified founder.

##### Number of known ancestors (NKA)

NKA_j_ is the total number of identified ancestors in the pedigree of individual j, within a specified number of generations.

##### Generational pedigree completeness (GPC)

GPC_j_ is defined as the mean CGE of all individuals in the target generation j divided by the ordinal number of that generation, expressed as a percentage.

##### Asymmetrical pedigree equivalent (APE)

APE_j_ quantifies the difference in recorded ancestral depth between the paternal and maternal lines of an individual j, and is computed in two complementary forms: cAPE, based on the complete generation equivalent, and mAPE, based on the maximum number of recorded generations. Full definitions are given in Sect.  Pedigree quality metrics: asymmetrical pedigree equivalent (APE) and pedigree tree imbalance (PTI).

##### Pedigree tree imbalance (PTI)

PTI quantifies the topological asymmetry of the pedigree in its entirety, treated as a rooted tree. The full definition is given in Sect.  Pedigree quality metrics: asymmetrical pedigree equivalent (APE) and pedigree tree imbalance (PTI). Together, APE and PTI capture two distinct aspects of pedigree imbalance: recording asymmetry between the parental lines and topological asymmetry of the entire pedigree.

#### A.4 Classical autosomal inbreeding coefficients

Three quantities together describe the inbreeding status of an autosomal allele in an individual. The classical inbreeding coefficient F [56, 57] gives the probability that the two alleles at a locus are identical-by-descent. Kalinowski’s new inbreeding coefficient F_N_ [57] restricts attention to first-time IBD events or autozygosity that did not exist in any ancestor. The partition F = F_N_ + F_aK_ separates first-time autozygosity from inherited ancestral autozygosity (the latter defined in A.5). Finally, the per-individual rate of inbreeding ΔF rescales F by pedigree depth, providing a quantity that is more comparable across individuals with unequal genealogical information. In González-Recio et al., (2007) ΔF has been suggested to detect inbreeding depression with greater sensitivity than F itself when pedigree depth varies substantially across individuals, although it is not yet routinely used in place of F.

##### Inbreeding coefficient (F)

F_j_, the classical inbreeding coefficient of an individual j, was originally developed by Wright [55] within the framework of path coefficient analysis. Its most intuitive definition was provided by Malécot [57], who defined it as the probability that two alleles randomly sampled at a locus in an individual are autozygous (IBD).

##### Kalinowski’s new inbreeding coefficient (F_**N**_).

F_Nj_ [58] is the probability that the two alleles at a locus in individual j are IBD for the first time in that individual, that is, their autozygosity originates from a pedigree loop in the current ancestry and was not previously IBD in any ancestor.

##### Individual increase in inbreeding (ΔF)

ΔF_j_ was proposed by González-Recio et al. [26] as an alternative inbreeding-related parameter that adjusts the individual inbreeding coefficient of an individual j for pedigree depth and reflects the rate of inbreeding accumulation in each animal. PedXylo computes ΔF_j_ according to the depth-corrected formulation of Gutiérrez et al. [27]: ΔF_j_ =1 − (1 − F_j_)^1/(CGE^_j_^− 1)^. As suggested [26] ΔF is less affected by pedigree depth and by nonlinear changes in inbreeding over time than F itself, and ΔF has been suggested to detect inbreeding depression with greater sensitivity than F. It may therefore be especially valuable in real pedigree datasets with substantial variation in pedigree completeness.

#### A.5 Ancestral inbreeding coefficients.

Classical inbreeding coefficients describe an individual’s current autozygosity. Ancestral inbreeding coefficients describe the history of that autozygosity — how often, and how far back, alleles have already passed through IBD states. The motivation is biological: alleles that have repeatedly been autozygous in ancestral generations have had repeated opportunities for selection against deleterious recessives and may therefore behave differently from alleles undergoing IBD for the first time.

##### Ballou’s ancestral inbreeding coefficient (F_**aB**_)

F_aBj_ [59] quantifies the extent to which alleles in an individual have been exposed to inbreeding in previous generations. Originally defined as the cumulative proportion of an individual’s genome that has undergone IBD in its ancestors, it is now commonly interpreted as the probability that an allele carried by an individual has experienced IBD at least once in its ancestral lineage [21], with subsequent methodological refinements improving its estimation (e.g. [30]). A more detailed formulation and recent developments are provided by Nomura [60].

##### Kalinowski's ancestral inbreeding coefficient (F_aK_)

F_aKj_ [58] is the probability that an allele which is currently autozygous (IBD) in an individual has already been autozygous at least once in its ancestral lineage. The classical inbreeding coefficient can be partitioned as

. F_j_ = F_Nj_ + F_aKj_, separating autozygosity arising for the first time from autozygosity inherited from previously inbred ancestors.

##### Ancestral history coefficient (AHC)

AHC_j_ [21] quantifies the cumulative history of ancestral autozygosity by measuring how many times, on average, an allele carried by individual j has been in an IBD state during its transmission through the pedigree. It rests on the premise that alleles that have experienced inbreeding more frequently in the past are less likely to be deleterious than alleles that have undergone IBD fewer times. This distinguishes AHC from F_aB_ and F_aK_, in which an allele contributes to ancestral inbreeding once it has undergone IBD at least once, regardless of how many times. For a particularly clear application, see Todd et al. [61]. Unlike the other coefficients defined as probabilities, AHC is not bounded between 0 and 1, because it counts repeated IBD events and may therefore exceed 1.

#### A.6 Partial inbreeding coefficients

The coefficients defined in A.4 and A.5 describe an individual’s inbreeding as a single number summed across all founders. Partial inbreeding coefficients [36] decompose that number by ancestor, asking what fraction of an individual’s autozygosity is attributable to each specific founder or ancestor. The decomposition is biologically motivated: if inbreeding depression varies among founders, because some carry more deleterious recessive variants than others, a single summed coefficient cannot detect this heterogeneity, but a partial-coefficient decomposition can. Partial coefficients are also useful in the management of small, closed populations, where they support the design of mating strategies that limit inbreeding contributions from specific influential ancestors.

##### **Partial inbreeding coefficient (**_**i**_**F**_**j**_**)**

_i_F_j_ is defined as the probability that individual j is autozygous (IBD) for a specified allele (numbered in gene-dropping process) inherited from a specified ancestor i [36]. PedXylo computes partial coefficients for all five inbreeding-coefficient families (F, F_N_, F_aB_, F_aK_, AHC) on both autosomes and the X chromosome, providing a comprehensive partial-decomposition framework. Of the publicly available pedigree-analysis programs that support partial coefficients - ENDOG [14], PEDIG [20], PyPedal [18], and PARTINBR [36] - each implements a more limited subset of these decompositions, generally for autosomal inheritance only.

#### A.7 Sex-linked inbreeding coefficients

Because of the sex-specific transmission of the X chromosome, X-linked inbreeding follows genealogical rules different from those of autosomal inheritance [24]. Outside the pseudoautosomal region, males are hemizygous and cannot be inbred at X-linked loci in the classical sense; females may. Fathers transmit their single X chromosome exclusively to daughters and never to sons, so X-linked identity-by-descent depends on the sequence of males and females in the pedigree. These features make sex-linked coefficients a distinct parameter family, not a simple modification of autosomal measures, and they are biologically relevant because recessive deleterious variants on the X chromosome may contribute to reduced fitness and may be exposed to sex-specific opportunities for selection and purging.

##### Sex-linked coefficients implemented in PedXylo

PedXylo computes a sex-linked counterpart for each autosomal coefficient defined above: the X-linked inbreeding coefficient (F_X_), Kalinowski's new sex-linked coefficient (F_NX_), the sex-linked individual increase in inbreeding (ΔF_X_), Ballou's ancestral sex-linked coefficient (F_aBX_), Kalinowski's ancestral sex-linked coefficient (F_aKX_), and the sex-linked ancestral history coefficient (AHC_X_). All sex-linked coefficients are additionally provided in their partial-decomposition forms.The classical sex-linked coefficients F_X_, F_NX_, and ΔF_X_ are not defined for hemizygous males, since autozygosity at a locus requires two alleles and males carry only one X chromosome outside the pseudo-autosomal region. PedXylo therefore applies a "relaxed" definition for the ancestral sex-linked coefficients F_aBX_, F_aKX_, and AHC_X_, which remain informative in males. Because a male's single X chromosome is inherited from his mother, these coefficients quantify the past autozygosity of the X-linked alleles he carries or the extent to which those alleles were exposed to a homozygous state in female ancestors. They therefore might be associated to hypotheses concerning the purging of X-linked recessive deleterious variants.

#### A.8 Effective population size

Effective population size (Ne) summarises the rate at which a population accumulates inbreeding due to its finite size or equivalently, the size of an idealised population that would produce the same rate of genetic drift as the population under study. Pedigree-based estimates of Ne are derived from the rate of inbreeding accumulation, ΔF, averaged across the reference population. Because inheritance at X-linked loci differs fundamentally from autosomal inheritance, X-linked Ne (Ne_X_) must be computed separately and may diverge substantially from autosomal Ne when sex-specific contributions, sex-biased migration, or sex-specific reproductive variance are present. Under idealised assumptions, X-linked effective population size is expected to approximate three-quarters of autosomal Ne [39]; departures from this expectation in real pedigrees are themselves informative.

##### Autosomal effective population size (Ne)

In diploid populations without self-fertilisation, PedXylo applies the depth-corrected formulation of Gutiérrez et al. [27]: the population-level rate of inbreeding ΔF (without individual subscript) is defined as the arithmetic mean of the individual increases in inbreeding (ΔF_j_; A.4) over all individuals of the target reference population, and the realised effective population size is estimated as Ne = 1 / (2ΔF).

##### Sex-linked effective population size (Ne_**X**_)

PedXylo extends the gene-dropping framework for inbreeding coefficients to sex-specific X-chromosome inheritance and combines this with the realised-effective-size logic based on the rate of inbreeding accumulation. Ne_X_ is therefore the size of an idealised population producing a rate of X-linked drift, expressed as ΔF_X_, equal to that observed in the study population. The population-level X-linked rate ΔF_X_ is defined analogously, as the arithmetic mean of ΔF_Xj_ across the females of the target reference population (ΔF_Xj_ is not defined for hemizygous males), and Ne_X_ is estimated as Ne_X_ = 1 / (2ΔF_X_). Pedigree-based Ne_X_ estimates may be particularly valuable as expectation-based comparators for genomic estimates, because the heterogeneous recombination landscape of the X chromosome means that genomic X-linked estimates may also reflect recombination suppression, linked selection, and restricted introgression rather than purely demographic processes [40].

## Declarations

### Content for publication

During the preparation of this work, the authors used Claude (Anthropic, Claude Opus 4.6) to assist with translating parts of the Python and Fortran90 codebase to Rust during software development, formatting references according to the Vancouver citation style, converting in-text citations from author-year to numbered format, and English editing of the final version of the manuscript and of the cover letter. The authors reviewed, verified, and edited all outputs and take full responsibility for the content of this publication.
